# Power and poison: The intersections of H_2_S and O_2_ metabolism

**DOI:** 10.1016/j.jbc.2025.110810

**Published:** 2025-10-13

**Authors:** Joseph Brake, Ruma Banerjee

**Affiliations:** Department of Biological Chemistry, University of Michigan, Ann Arbor, Michigan, USA

**Keywords:** hydrogen sulfide, oxygen metabolism, hypoxia, hypoxia-inducible factor, gut microbiome, fumarate, gut dysbiosis, butyrate

## Abstract

The metabolic interaction between hydrogen sulfide (H_2_S) and oxygen (O_2_) exemplifies the interplay between chemical power and poison at the electron transport chain as these gases influence the conversion of nutrient energy to cellular currency. H_2_S is a product of mammalian and microbial metabolism and is both an inorganic nutrient and a respiratory poison. In its former role, H_2_S transfers its reducing power to coenzyme Q as it is oxidized by sulfide quinone oxidoreductase in the inner mitochondrial membrane. As a respiratory poison, H_2_S inhibits complex IV and profoundly influences intracellular O_2_ levels with pleiotropic effects on hypoxia sensing and signaling, and on cellular metabolism, glimpses of which are only just beginning to emerge. The high concentration of luminal sulfide in the lower gut, combined with the steep radial O_2_ gradient, ranging from a virtually anoxic lumen to a highly vascular lamina propria, raises many questions about how the interaction between these gases plays out with local and long-range impacts on biology. Their interaction is equally germane in other hypoxic tissues where endogenous H_2_S production and/or constitutively low-sulfide oxidation capacity could potentially dial up O_2_ availability. Importantly, H_2_S oxidation can prevail even when its concentration rises to levels that poison complex IV and is enabled by rerouting electrons through complex II, using fumarate as a terminal electron acceptor. Methodological advancements that support the quantitative analysis of *in vivo* models will be critical for broadening our understanding of the metabolic and physiological import of the O_2_–H_2_S interplay.

Sulfide chemistry is inextricably tied to cyanosulfidic protometabolism and to the chemical stirrings of life that started during or after abatement of meteoritic bombardments, creating conditions that were conducive for hydrogen cyanide generation. Subsequent synthesis of precursors of extant biomolecules is postulated to have occurred *via* reductive homologation of hydrogen cyanide and its derivatives, fueled by UV light and hydrogen sulfide (H_2_S) (1). The great oxygenation event, some 2.3 billion years ago (2), was cataclysmal for the anaerobic biosphere and impelled the expansion of prevailing metabolic networks and the evolution of enzymes that utilized oxygen (O_2_) as well as those that mitigated its biotoxicity (3). As life forms diversified in oceanic waters that were initially rich in sulfide but poor in O_2_ (4), a transition to an O_2_-rich but sulfide-poor atmosphere ensued. A superanoxic shift driven by rising H_2_S and falling O_2_ is postulated to have driven the Permian–Triassic mass extinction some 500 million years ago and slowed down subsequent recovery (5).

Today, only a limited number of anoxic or near-anoxic niches still harbor micro-organisms that use H_2_S as an electron source, such as green sulfur (S) bacteria. Yet, even in complex animals, the specter of an ancient sulfide-rich world remains. The mitochondrion likely arose from an endosymbiotic event ∼1.5 billion years ago, when environmental H_2_S was high. It is hypothesized that the original endosymbiont contained a sulfide-oxidizing enzyme, sulfide quinone oxidoreductase (SQOR), which was likely essential for its survival (6). Today, mammalian SQOR couples sulfide oxidation to coenzyme Q reduction and provides electrons to the mitochondrial electron transport chain (ETC), using O_2_ as the terminal electron acceptor (7, 8, 9, 10) (Fig. 1). This chemolithotrophic oxidation of sulfide powers ATP generation (11) when H_2_S levels are low (12), but poisons complex IV when H_2_S levels rise (12, 13, 14), increasing intracellular O_2_ levels (15).

**Figure 1:**
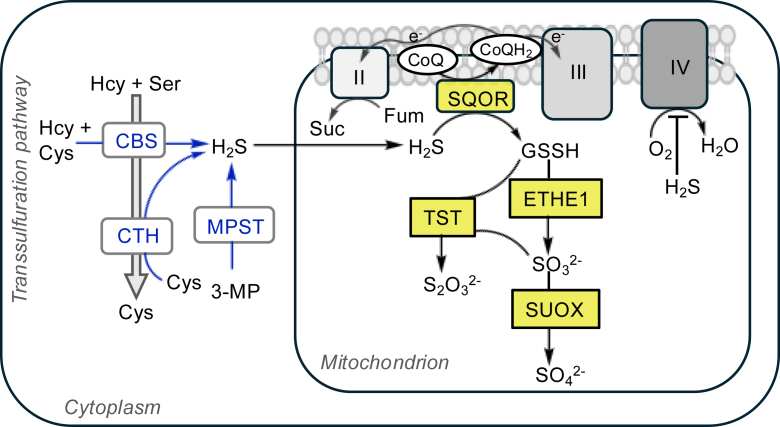
**H_2_S and O_2_ metabolism intersect at the ETC.** H_2_S is produced by the *trans*-sulfuration pathway enzymes CBS and CTH from cysteine and/or homocysteine (Hcy) or from 3-mercaptopyruvate (3-MP) by MPST (*blue arrows*). The mitochondrial sulfide oxidation pathway (*yellow boxes*) oxidizes H_2_S as CoQ is reduced by SQOR in the first step. Glutathione persulfide (GSSH) generated in this step is further oxidized to either thiosulfate or sulfate by ETHE1, TST, and SUOX. Electrons from CoQH_2_ typically proceed to complex IV driving O_2_ consumption but are redirected to complex II when complex IV is inhibited or O_2_ is limiting. Complex II uses fumarate (Fum) as a terminal electron acceptor, reducing it to succinate (Suc). CBS, cystathionine β-synthase; CoQ, coenzyme Q; CTH, γ-cystathionase; ETC, electron transport chain; ETHE1, persulfide dioxygenase; H_2_S, hydrogen sulfide; MPST, mercaptopyruvate sulfurtransferase; O_2_, oxygen; SQOR, sulfide quinone oxidoreductase; SUOX, sulfite oxidase; TST, thiosulfate sulfur transferase.

The lower gut is a nearly anoxic refuge settled by a multitude of O_2_-averse micro-organisms. Sulfate-reducing gut bacteria such as *Desulfovibrio* generate copious quantities of H_2_S, ultimately derived from cysteine catabolism (16, 17, 18). H_2_S is also a product of mammalian metabolism, generated primarily from cysteine *via* the actions of cystathionine β-synthase (CBS), γ-cystathionase, and mercaptopyruvate sulfurtransferase (19, 20) (Fig. 1). Gut sulfide metabolism is a quantitatively significant contributor to systemic host sulfide metabolism, and colonocytes are adapted to high luminal sulfide by virtue of high SQOR expression (12). The complete oxidation of sulfide to sulfate involves reactive sulfur species intermediates, including persulfide, polysulfide, and thiosulfate (21) (Fig. 1). Reduction of persulfides and polysulfides releases H_2_S that can cycle back to the sulfide oxidation pathway (22). Importantly, persulfidation of cysteine residues on proteins protects the proteome from irreversible overoxidation (23).

The intersection of H_2_S and O_2_ at the ETC (Fig. 1) underlies their dynamic metabolic interactions, which remain poorly characterized. This review explores the role of H_2_S in modifying cellular O_2_ levels and the threshold for O_2_ sensing. It also examines the interplay between high H_2_S and severe hypoxia in the gut with potential therapeutic implications for mitochondrial function disorders.

## Regulation of hypoxia-inducible factor–dependent O_2_ sensing by H_2_S

**Figure 2:**
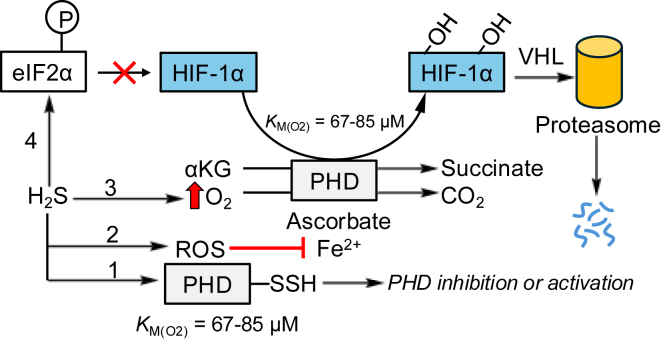
**Alternative possibilities for HIF regulation by H_2_S.** HIF-1α undergoes rapid proteasomal degradation through PHD-mediated proline hydroxylation, which is inhibited under hypoxic conditions. Mechanistic alternatives postulated for H_2_S-dependent HIF regulation are (1) persulfidation of PHD, leading to its activation or inhibition, (2) ROS-dependent oxidation of Fe^2+^ in PHD, (3) substrate-level activation of PHD *via* increased O_2_ availability, and (4) global translation inhibition *via* eIF2α phosphorylation. eIF2α, eukaryotic initiation factor 2α; HIF, hypoxia-inducible factor; H_2_S, hydrogen sulfide; O_2_, oxygen; PHD, prolyl hydroxylase; ROS, reactive oxygen species; VHL, von Hippel-Lindau protein.

Prolyl hydroxylases (PHDs) catalyze the hydroxylation of proline residues in the α-subunit of hypoxia-inducible factor (HIF) (24), marking it for ubiquitination by von Hippel-Lindau tumor-suppressor protein and subsequent proteasomal degradation (Fig. 2) (25, 26). PHDs are effective O_2_ sensors by virtue of their *K*_*M*_ for O_2_ being 67 to 85 μM (corresponding to 6–8% O_2_) (27), which renders them sensitive to O_2_ fluctuations within a physiologically relevant concentration range. As O_2_ levels fall and PHD activity becomes limiting, HIF α and β subunits heterodimerize to activate an adaptive transcriptional response (28).

There are conflicting reports in the literature as to whether H_2_S stabilizes or destabilizes HIF. The underlying mechanisms that have been proposed to explain the effects of H_2_S on HIF include (1) persulfidation of PHD (29, 30), (2) reactive oxygen species (ROS)–dependent inhibition of PHD (31), (3) substrate-level activation of PHD (15), and (4) global translation inhibition (32) (Fig. 2, *paths* 1–4). Hypoxic HIF-1α stabilization was reportedly impeded by the slow-releasing sulfide donor GYY4137, and by inorganic polysulfides, but surprisingly, also by knockdown of CBS, an endogenous source of both H_2_S and persulfide (29). A different study reported the opposite effect of H_2_S and GYY4137, that is, rescue of HIF destabilization by CBS inhibition (30). In both studies, PHD persulfidation (Fig. 2, *path 1*) was invoked as the underlying mechanism, albeit not rigorously examined.

A third study reported HIF-1α stabilization by H_2_S, which was, however, dependent on xanthine oxidase, an ROS producer (31). The direct role of ROS in H_2_S-dependent modulation of HIF is important to consider since active PHD activity requires the mononuclear iron (Fe) cofactor to be in the ferrous state (Fig. 2, *path 2*). Simulating superoxide production at the Q_0_ site in complex III reportedly increases HIF signaling, whereas inhibiting ROS generation at the same site elicits the opposite effect (33). As H_2_S levels rise (34) or O_2_ levels drop (35), electrons are rerouted away from complex III and toward complex II (Fig. 1). While ROS production at the Q_0_ site in complex III increases under hypoxia and contributes to HIF-1α stabilization (36), it is unclear how H_2_S impacts ROS generation at this site.

Inhibition of global eukaryotic initiation factor 2α (eIF2α)–mediated protein translation has been invoked as yet another mechanism of hypoxic HIF destabilization by H_2_S (32) (Fig. 2, *path 4*), which is consistent with a report that H_2_S increases phospho-eIF2α levels *via* persulfidation of protein phosphatase 1c (37). However, a different study ruled out H_2_S-mediated HIF destabilization through translation inhibition (38). Thus, while phospho-eIF2α-dependent repression of general translation decreases HIF, which turns over rapidly (39), it is unclear to what extent this mechanism contributes to its destabilization by H_2_S.

The potential to regulate PHD by influencing O_2_ availability derives from the ability of H_2_S to both stimulate and inhibit O_2_ consumption, depending on its concentration (11, 13). We have recently quantified the H_2_S dose-dependent increase in intracellular O_2_ across several cell lines using a fluorescent hypoxia sensor (15) (Fig. 2, *path 3*). Our study revealed the capacity of H_2_S to profoundly influence intracellular O_2_ such that cells grown in the presence of 2% O_2_ and 25 or 100 ppm H_2_S respond to the equivalent of 5% to 9% or 13% to 17% O_2_ exposure, respectively, and destabilize HIF-1α. A previous study also reported transient destabilization of HIF in response to bolus administration of Na_2_S, which was shown to be von Hippel-Lindau dependent and correlated with decreased O_2_ consumption (38). The extent to which the conflicting reports are dependent on the experimental contexts is an important issue that merits resolution in the study of H_2_S regulation of HIF signaling.

## Effect of H_2_S on other O_2_-dependent enzymes

**Figure 3:**
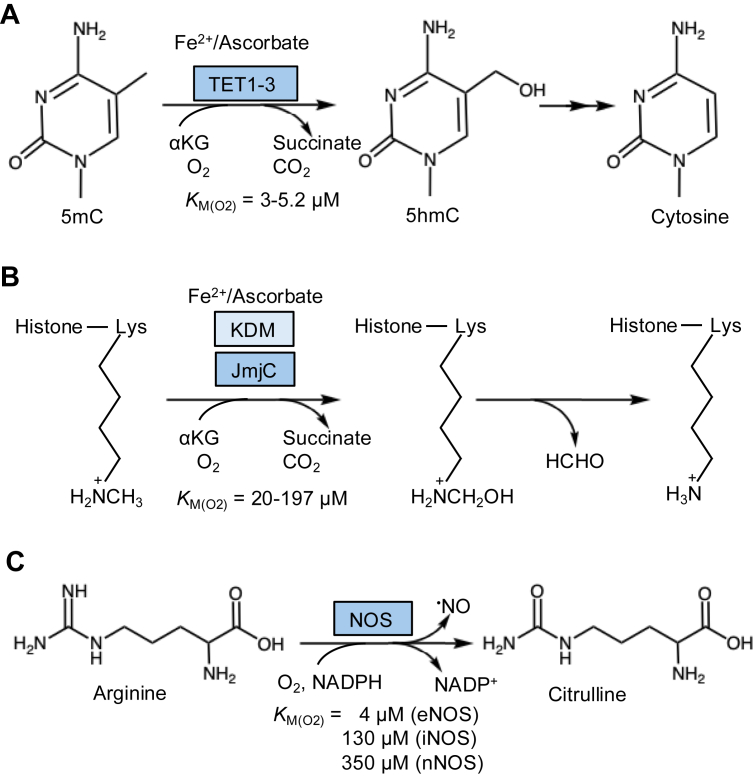
**O_2_-dependent enzymes that are expected to be sensitive to O_2_ modulation by H_2_S.***A*, TET enzymes are α-ketoglutarate (αKG)-dependent DNA demethylases that hydroxylate methylcytosine and exhibit a low *K*_*M*_ for O_2_. *B*, JmjC family lysine demethylases (KDMs) are also αKG-dependent but have a higher *K*_*M*_ for O_2_ than the TET enzymes. KDMs catalyze the formation of a hydroxymethyl amine intermediate, which is subsequently demethylated to lysine. *C*, nitric oxide synthase (NOS) is a mono-oxygenase that synthesizes ^•^NO from arginine. The *K*_*M*_ for O_2_ depends on the NOS isoform. eNOS, epithelial NOS; H_2_S, hydrogen sulfide; iNOS, inducible NOS; JmjC, Jumonji C; KDM, lysine demethylase; ^•^NO, nitric oxide; nNOS, neuronal NOS; O_2_, oxygen; TET, ten–eleven translocation.

An increase in intracellular O_2_ by H_2_S has implications for other cellular outputs that rely on O_2_. While >200 enzymes in humans either use or are likely to use O_2_ as a substrate, a key consideration for sensitivity to fluctuations in ambient O_2_ levels is whether the *K*_*M*_ for O_2_ is in a physiologically relevant concentration range (40). From a recent compilation of candidate O_2_ sensors (40), a subset that might be influenced by H_2_S is discussed below (Fig. 3).

The TET (ten–eleven translocation) enzymes are 2-ketoglutarate-dependent dioxygenases that convert DNA 5-methylcytosine to 5-hydroxymethylcytosine, facilitating subsequent cytosine demethylation (41) (Fig. 3*A*). TETs are important epigenetic regulators that promote gene activation by reversing CpG methylation, which typically silences genes. Their low *K*_*M*_ for O_2_ (3.0–5.2 μM or 0.31–0.53% O_2_ (42)) suggests that TETs would only be sensitive to changes in ambient O_2_ under severely hypoxic conditions. H_2_S reportedly promotes TET1 and TET2 expression in T cells (43) and elicits genome-wide (up and down) changes in DNA CpG methylation in fish adapted to life in sulfide-rich relative to nonsulfidic waters (44). Interestingly, H_2_S-induced TET-mediated CpG island demethylation of the Klotho promoter results from amelioration of renal hypoxia in a murine model for chronic kidney disease (45). In this model, chronic hypoxia enhanced Klotho promoter methylation, silencing expression of the encoded renoprotective protein. In a physiological context, H_2_S could enhance O_2_ availability *via* vasodilation, increasing blood flow and O_2_ delivery, as well as by decreasing intracellular O_2_ consumption by the ETC. The susceptibility of TET enzymes, which rely on mononuclear ferrous Fe for activity, to ROS-mediated inactivation might also be exacerbated by hypoxia and attenuated by H_2_S (45).

A second O_2_-sensitive epigenetic regulator is the Jumonji C domain–containing family of lysine demethylases (KDMs), which, like the TET enzymes, are also 2-ketoglutarate-dependent dioxygenases (40) (Fig. 3*B*). Twenty-three of the 32 annotated Jumonji C proteins in humans catalyze lysine demethylation of histone and nonhistone substrates. The *K*_*M*_ for O_2_ varies over a 10-fold range (∼20–200 μM corresponding to ∼2–20% O_2_) for KDMs, with some of the reported variability resulting from differences in assay conditions (40). The capacity for regulating KDMs by H_2_S is poorly understood despite the potentially consequential impact on the epigenome and other cellular functions such as mitochondrial biogenesis *via* KDM3A-dependent Lys-224 demethylation of PGC-1α (46) and KDM5C-dependent autophagy induction by Arg-170 demethylation of ULK1 (47). H_2_S reportedly increases PGC-1α persulfidation and can inhibit or upregulate its expression, with these contradictory results being obtained with endogenous *versus* exogenous modulation of sulfide levels (48). Decreased persulfidation is correlated with increased ULK1 activity (49). The effects of H_2_S on PGC-1α and ULK1 methylation status have, however, not been examined.

Nitric oxide synthase (NOS) is a mono-oxygenase with a *K*_*M*_ for O_2_ that is reported to be 350 μM for neuronal NOS (50), 130 μM for inducible NOS (51), and 4 μM for endothelial NOS (52) (Fig. 3*C*). The varying *K*_*M*_ values might reflect distinct roles for the NOS isoforms (53). Like H_2_S, nitric oxide (^•^NO) is a reversible inhibitor of complex IV (54), and inhibitory concentrations of ^•^NO increase intracellular O_2_ and destabilize HIF (55). Interestingly, ^•^NO stimulates 2-aminoethanethiol dioxygenase–catalyzed proteolysis of N-degrons by increasing O_2_ availability (56). ^•^NO stimulates H_2_S synthesis by reprogramming the *trans*-sulfuration pathway (57, 58), whereas H_2_S reportedly increases ^•^NO by upregulating endothelial NOS expression during chronic tissue hypoxia (31). Although a multitude of mechanisms have been proposed (31), the role of O_2_-dependent regulation of ^•^NO synthesis by H_2_S has not been considered.

H_2_S stimulates lipid biogenesis (59) and increases lipid oxidation in cells (15). It is possible that stimulation of lipid oxidation by H_2_S contributes to the group of nonenzymatic oxidative reactions that help maintain a deeply anaerobic gut lumen in both germ-free and conventional mice (60). H_2_S and O_2_ chemistry also intersect at globin-bound ferric hemes, which coordinate and oxidize sulfide to thiosulfate and ferrous heme–bound catenated polysulfur species in hemoglobin (61, 62), myoglobin (63), and neuroglobin (64). Based on the bimolecular rate constants for sulfide binding to ferric heme, globins are expected to play a minor role in O_2_ metabolism linked to H_2_S oxidation.

## H_2_S regulates mitochondrial O_2_ consumption

Mitochondrial respiration functions as an O_2_ sink and helps establish intracellular O_2_ gradients (65, 66, 67, 68). The absence of functional complex IV in SCO2^−/−^ cells leads to intracellular O_2_ accumulation concomitant with oxidative DNA damage (69). The perinuclear localization of mitochondria helps shield genomic DNA from the genotoxicity associated with O_2_ hyperaccumulation (70). Destabilization of Fe–S cluster proteins, a hallmark of intracellular hyperoxia (71), is seen in cells cultured with chronic H_2_S exposure as well as in the colon of Villin^Cre^SQOR^fl/fl^ mice in which SQOR was knocked down in intestinal epithelial cells (15).

Hyperoxia is a contributing factor to mortality in mitochondrial diseases. Mitochondrial myopathy patients have elevated venous pO_2_ and blood lactate after aerobic forearm exercise, indicating systemic O_2_ accumulation (72). In a mouse model of mitochondrial disease lacking the complex I subunit Ndufs4, breathing air containing 11% instead of 21% O_2_ from day 30 prevented brain lesions and neurological symptoms and improved survival from 58 to 270 days (73, 74). In contrast, breathing 55% O_2_ was lethal within 2 to 8 days of exposure in the same mice (73, 74). Pharmacological mimicry of hypoxia using a combination of GBT440 (to increase the O_2_ binding affinity of hemoglobin) and PT2399 (to inhibit HIF-2α), extended lifespan and protected against neurological defects in Ndufs4 KO mice (75). These findings demonstrate the potential therapeutic utility of hypoxia for mitochondrial diseases (75).

In humans, inhalation of 10 ppm H_2_S impaired respiration during acute exercise with lowered VO_2_ and elevated blood lactate (76), whereas ≤5 ppm H_2_S was without effect (77). Oxidation by SQOR is critical for countering sulfide toxicity (12). SQOR activity can also be supported by complex II, using fumarate as a terminal electron acceptor (Fig. 1) (34). While the quantitative significance of complex II-dependent sulfide oxidation by SQOR to whole body sulfide clearance is not known, O_2_ accumulation is also predicted to occur when this additional disposal route is activated because of inhibition of complex IV (34, 78). We speculate that increased O_2_ availability in skeletal muscle contributes to improved exercise endurance in rodents by the natural product ergothioneine, which increases endogenous H_2_S biosynthesis (79, 80).

SQOR-mediated H_2_S oxidation capacity can affect tissue-level respiratory responses to H_2_S. In ground squirrels, brain SQOR expression is 100-fold higher than in other rodents like mice and rats and protects them against ETC inhibition by exogenous sulfide or hypoxia-induced H_2_S (81). Furthermore, seasonal fluctuations in hepatic SQOR activity in hibernating ground squirrels are correlated with low respiration rate during torpor when SQOR activity is also low (82).

Hereditary SQOR deficiency presents as Leigh syndrome (83), a mitochondrial disease characterized by severe neurological and neuromuscular defects (84). Murine models of whole-body SQOR knockdown exhibit stunted growth in early life and death by 8 to 10 weeks of age (81, 85). A low sulfur diet, or administration of antibiotics to mitigate gut microbial sulfide production, normalized blood lactate, prolonged lifespan, increased body temperature, and prevented motor dysfunction in SQOR-deficient mice, concomitant with lowered H_2_S and elevated complex IV activity in brain, muscle, and liver (86). It is possible that a hyperoxic shift is a contributing factor to the etiology of defects at other loci in the sulfide oxidation pathway, for example, ETHE1 (87) and sulfite oxidase (88), which are correlated with elevated H_2_S.

In colon epithelial cells, SQOR expression levels influence the O_2_ consumption rate (OCR) (12, 54, 89). Furthermore, varying SQOR expression levels render tissues differentially susceptible to the toxic effects of H_2_S. Colon epithelial cells are adapted to chronic exposure to luminal H_2_S by high SQOR expression (12). Other tissues that have high SQOR expression include skeletal muscle, liver, and lung (https://www.proteinatlas.org/ENSG00000137767-SQOR/tissue). On the other hand, the brain exhibits low SQOR expression and is sensitive to O_2_ deprivation. Neuron-specific SQOR overexpression in mice mitigated hypoxic H_2_S accumulation, improved survival under severe hypoxia, and protected against injury and death following cerebral ischemia (81, 90). Besides the brain, other tissues that are expected to be sensitive to H_2_S exposure include the ovary, testis, and heart muscle because of low SQOR expression, as well as the retina, where its mRNA expression is low (https://www.proteinatlas.org/ENSG00000137767-SQOR/tissue).

## H_2_S and O_2_ in gut

The lower gut is characterized by a steep radial O_2_ gradient ranging from an O_2_-rich subepithelial mucosa to a nearly anoxic lumen (91). Gut microbes are adapted to an anoxic environment and exhibit varying tolerance to elevated luminal O_2_, which in turn affects host–microbiome interactions (92, 93). Unlike obligate anaerobes that are O_2_ intolerant, facultative anaerobes are resistant to an increase in luminal oxygenation (94). Obligate anaerobes such as Clostridia and Bacteroida ferment complex carbohydrates to short-chain fatty acids like butyrate, propionate, and acetate, which support host epithelial cell metabolism (94). In contrast, facultative anaerobes such as Enterobacteriaceae do not consume fiber, interfere with host nutrition, and are considered to be dysbiotic. A broad O_2_-dependent shift in microbiome composition from obligate to facultative anaerobes is observed in many chronic human illnesses (95).

Sulfate-reducing bacteria are obligate anaerobes that contribute to gut H_2_S synthesis and exhibit increased relative abundance in Crohn’s disease (96, 97). However, a study on pediatric patients with Crohn’s disease reported that increased fecal H_2_S was correlated with decreased chronic inflammation (98). An increase in the abundance of sulfate-reducing bacteria has also been implicated in ulcerative colitis (99) and colorectal cancer (100). Mucolysis *via* the addition of sulfide across disulfide bonds in mucin is postulated to underlie epithelial injury and inflammation (101). We posit that sulfide-mediated increase in O_2_ because of respiratory inhibition could also be a contributing factor, since hyperoxia incites gut injury and triggers dysbiosis (102, 103). To solidify a possible mechanistic link between H_2_S and hyperoxia in bowel diseases, studies with colon expression of O_2_ reporters in mice exposed to varying H_2_S *via* genetic or dietary modulation will be important.

**Figure 4:**
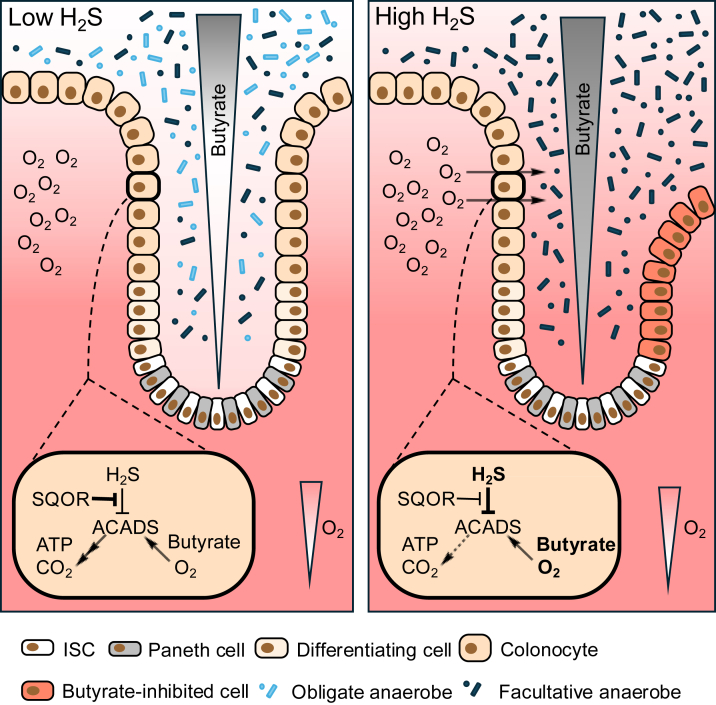
**H_2_S and O_2_ interactions predicted at the host–microbiome interface in colon.***Left*, H_2_S concentrations that do not exceed SQOR oxidation capacity support the metabolism of microbiome-derived butyrate by ACADS in the fatty acid oxidation pathway, consuming O_2_ and yielding ATP. SQOR activity, which is preferentially localized at crypt apices, helps create a butyrate gradient down the crypt length and shields the lumen from O_2_, originating in the vascular lamina propria, and protects intestinal stem cells (ISCs) from butyrate. *Right*, predicted consequences of high H_2_S exposure in gut, which outstrips the oxidation capacity of SQOR. Inhibition of complex IV (by H_2_S) and ACADS (by SQOR-dependent CoA persulfide synthesis) leads to a buildup of butyrate and O_2_, diminishing the butyrate gradient and oxygenating the lumen. These changes promote luminal expansion of dysbiotic facultative anaerobes, leading to the loss of microbiome diversity and impair ISC differentiation *via* butyrate-mediated HDAC inhibition. ACADS, acyl-CoA dehydrogenase; CoA, coenzyme A; HDAC, histone deacetylase; H_2_S, hydrogen sulfide; O_2_, oxygen; SQOR, sulfide quinone oxidoreductase.

Stem cells reside at the base of crypts and differentiate into mature colonocytes and specialized postmitotic cells, including goblet, Paneth, and enteroendocrine cells (91) (Fig. 4, *left*). Differentiating cells migrate from the base of the crypt to the surface, where they turn over every 3 to 5 days (104). Disruption of crypt stem cell differentiation and migration is a hallmark of bowel diseases, leading to inflammation and tissue damage (105). Sulfide oxidation pathway enzymes are concentrated at the apices of crypts, that is, at the host–microbiome interface (12).

At a cellular level, sulfide concentrations below ∼20 μM stimulate OCR, whereas concentrations ≥30 μM inhibit it in the human colorectal adenocarcinoma cell line, HT-29 (12). SQOR knockdown sensitizes these cells to respiratory poisoning at sulfide doses as low as 5 μM (12). Another study similarly reported that HT-29 cells exhibit decreased OCR at >40 μM H_2_S but increased OCR at 20 μM, which was further enhanced by butyrate (106). The biphasic effect of H_2_S on OCR is illustrative of how luminal H_2_S is broadly toxic to the gut at high concentrations but beneficial at low concentrations (107, 108, 109, 110) and suggests that H_2_S-mediated O_2_ regulation can influence gut health.

Short-chain fatty acids derived from microbial metabolism account for 60% to 70% of cellular energy requirements in colonocytes (111, 112). Butyrate consumption in the murine cecum decreases O_2_ levels and stabilizes HIF (113). Depletion of butyrate-producing *Clostridia* by antibiotic treatment or by *Salmonella* virulence factors increases epithelial oxygenation and drives pathogen expansion (114). Diminished butyrate production in the setting of intestinal dysbiosis leads to decreased epithelial respiration, which in turn contributes to increased luminal O_2_ (115), exemplifying a reciprocal metabolic relationship between these two substrates. Butyrate utilization by epithelial cells at crypt apices is important for limiting exposure in crypt bases, since butyrate has an antiproliferative effect on intestinal stem/progenitor cells, which is mediated by inhibition of histone deacetylase (116). Like other rapidly dividing cells, stem cells rely primarily on aerobic glycolysis and exhibit low respiratory capacity relative to epithelial cells (117). We predict that excess H_2_S originating from host or microbial metabolism interferes with epithelial butyrate consumption by inhibiting respiration and impairs the protective butyrate gradient along the crypt length, decreasing stem cell regeneration (Fig. 4, *right*). The pleiotropic consequences of these changes might include inflammation and leaky gut.

H_2_S inhibits short-chain acyl-CoA dehydrogenase (ACADS) in the beta-oxidation pathway (118). A potential mechanism for this inhibition is *via* SQOR-mediated formation of CoA persulfide, a potent ACADS inhibitor (119) (Fig. 4). Increased synthesis of CoA persulfide could exert an inhibitory effect on respiration by lowering flux through the beta-oxidation pathway. An increase in luminal H_2_S might represent a physiological setting in which SQOR-mediated CoA persulfide synthesis serves to prioritize oxidation of H_2_S over butyrate. While the *k*_cat_/*K*_*M*_ for CoA is twofold higher than for GSH, the strategy for substrate switching from the more abundant GSH to CoA or the mechanism by which ACADS inhibition by CoA persulfide is reversed are unclear. Since CoA persulfide is a tight-binding inhibitor of ACADS (120), the half-life of its inhibitory effect could impact O_2_ consumption even after H_2_S levels have dropped. However, H_2_S itself can affect ETC flux *via* prolonged fractional inhibition of complex IV (14). How the dual effects of H_2_S on attenuating butyrate oxidation at the level of ACADS and decreasing ETC flux at the level of complex IV are balanced *in vivo* awaits elucidation. As noted previously, sulfide oxidation can continue even when complex IV is inhibited *via* the use of fumarate as a terminal electron acceptor (Fig. 1). Restoration of butyrate consumption in colonocytes following SQOR-mediated sulfide clearance could be instrumental for deoxygenation, supporting short-chain fatty acid production by anaerobic microbes. Further *in vivo* studies will be critical for evaluating colonocyte SQOR as a therapeutic target in bowel diseases characterized by dysregulated H_2_S and O_2_.

## Conclusion and perspectives

**Figure 5:**
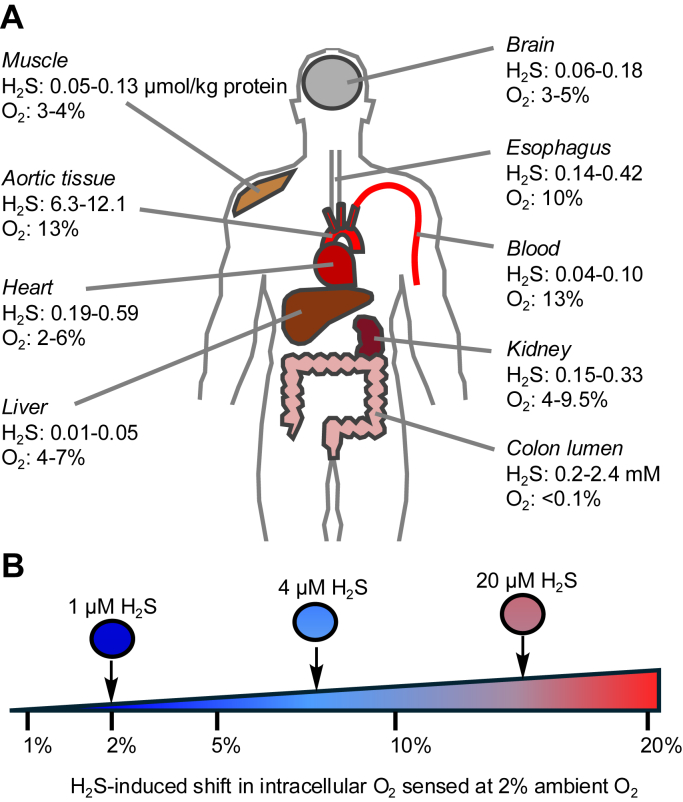
**H_2_S and O_2_ levels in mammalian tissues and cells.***A*, tissue levels of free H_2_S (μmol/kg protein except in colon lumen where the unit is specified) and O_2_ (percent of O_2_, converted from pO_2_ values in mm Hg). The tissue concentrations are from the indicated citations: colon H_2_S (16, 17), other tissue H_2_S (121), colon O_2_ (92), esophagus O_2_ (122), muscle O_2_ (123), other tissue O_2_ (40). The aortic O_2_ concentration is extrapolated from the value for blood where O_2_ concentration is primarily determined by hemoglobin concentration and O_2_-binding capacity. *B*, schematic showing perturbation in intracellular O_2_ sensed by cells cultured in an atmosphere of 2% O_2_ admixed with varying concentrations of H_2_S (10, 25, or 100 ppm), corresponding to 1, 4, or 20 μM dissolved H_2_S in the medium. O_2_ levels were estimated relative to 2% ambient O_2_ without H_2_S (15). H_2_S, hydrogen sulfide; O_2_, oxygen.

In many mammalian tissues, free H_2_S concentrations are below 0.1 μmol/kg protein, and O_2_ levels are 2% to 10%, with a major exception being the colonic lumen, where H_2_S is estimated to be 0.2 to 2.4 mM and O_2_ <0.1% (Fig. 5*A*) (16, 17, 40, 92, 121, 122, 123). Emerging evidence that chronic exposure to even low levels of H_2_S has the capacity to profoundly increase the intracellular O_2_ budget (Fig. 5*B*) has far-reaching implications for aerobic metabolism, particularly at the host–microbiome interface. In this setting, sulfide homeostasis itself is a collaborative production of host and microbial metabolism. A critical current limitation in the field is the frequent extrapolation from cellular studies to the complex gut environment with many unknowns, including the contribution of the disulfide-rich mucin layer to shielding host cells from sulfide. In fact, the redox state of the mucin layer could be an important contributor to the interplay between H_2_S and O_2_, influencing gut health by serving as a sulfide sponge. Strategies to evaluate changes in mucin redox status in health and in response to perturbations in microbial composition and host metabolism represent an important unmet need in the field.

While this review focused on the H_2_S–O_2_ metabolic interplay between host and the microbiome, dietary modulation represents a critical third arm in this interaction troika. In a mouse model for SQOR deficiency in intestinal epithelial cells, a 2.5-fold increase in dietary methionine, a metabolite that is upstream of the H_2_S-producing *trans*-sulfuration pathway, induced multiple changes (124). The local effects included degenerated crypt architecture and a shift in the gut microbiome composition. The long-range effects included a systemic change in energy metabolism, including a shift toward ketogenesis, and behavioral changes, including lower exploratory locomotor activity, which was correlated with brain pathology (124). The study highlighted the profound potential for pleiotropic impacts in the host–microbiome–diet trine and its sensitivity to genetic and nutrient perturbations. The physiological ramifications of these interactions in the context of H_2_S and O_2_ metabolism, emanating from the gut and impacting distant organs like the brain, are rich and virtually unexplored areas of studies. Methodological advances that enable a more *in vivo* focus will be critical for deepening our understanding of the power *versus* poison interplay between H_2_S and O_2_ in physiology.

## Dedication

R. B. dedicates this review to the fond memory of Bill Smith, who she is grateful to have known as a mentor, colleague, and friend, and who was instrumental in inviting her into the *Journal of Biological Chemistry* community.

## Conflict of interest

R. B. is a consultant for Zyphore Therapeutics Inc. All other authors declare that they have no conflicts of interest with the contents of this article.
